# Low-Value Care: Convergence and Challenges: Comment on "Key Factors That Promote Low-Value Care: Views From Experts From the United States, Canada, and the Netherlands"

**DOI:** 10.34172/ijhpm.2022.7017

**Published:** 2022-11-02

**Authors:** Sara Ingvarsson, Per Nilsen, Henna Hasson

**Affiliations:** ^1^Department of Learning, Informatics, Management and Ethics, Karolinska Institutet, Stockholm, Sweden.; ^2^Department of Health, Medical and Caring Sciences, Linköping University, Linköping, Sweden.; ^3^Unit for implementation and evaluation, Center for Epidemiology and Community Medicine (CES), Stockholm, Sweden.

**Keywords:** Low-Value Care, De-implementation, Overuse, Overtreatment, Overdiagnosis, Disinvestment

## Abstract

Interest has increased in the topic of de-implementation, ie, reducing so-called low-value care (LVC). The article "Key Factors That Promote Low-Value Care: Views From Experts From the United States, Canada, and the Netherlands" by Verkerk and colleagues identifies national-level factors affecting LVC use in those three countries. This commentary raises three critical points regarding the study. First, the study does not clearly define the national level. Secondly, national-level factors might not be relevant for all types of LVCs and thirdly, the study’s rather limited sample makes it difficult to draw firm conclusions. We also include some critical comments related to some of the study’s findings in relation to results of our recently published scoping review of the international literature on de-implementation and use of LVC and an interview study with primary care physicians on LVC use. Finally, we provide some suggestions for further research that we believe is needed to improve understanding of LVC use and facilitate its de-implementation.

## Introduction

Interest has increased in the topic of de-implementation—ie, reducing so-called low-value care (LVC).^1^ In their highly interesting article “Key Factors That Promote Low-Value Care: Views From Experts From the United States, Canada, and the Netherlands,” Verkerk et al^2^ identify national-level factors affecting LVC use in those three countries. Based on interviews with 18 experts, they describe the relevance of three types of factors in LVC usage: systemic factors (payment structure, industry, and malpractice litigation), knowledge factors (evidence and medical education), and social factors (public culture and medical culture).

Verkerk et al^2^ make highly relevant contributions to the ongoing discussion and knowledge accumulation concerning LVC use, and the study has many merits. However, we also have some reservations about some aspects of the study. Therefore, we begin our commentary by raising three critical points regarding the study as a whole before we address some of the study’s findings in relation to results of our recently published scoping review of the international literature on de-implementation and use of LVC^3^ and an interview study with primary care physicians on LVC use.^4^ Finally, we provide some suggestions for further research that we believe is needed to improve understanding of LVC use and facilitate its de-implementation.

## Overall Critique of the Study

We would like to raise three main critical points in relation to the study. First, the study does not clearly define the national level. Nations differ substantially with regard to how they govern healthcare; some countries have more national-level governance while others rely more on local-level steering. For instance, in Sweden, the national level consists of producing guidelines, health technology assessment and decisions on which treatments should be covered by the national insurance, whereas most of the financial system is locally governed in each of the 21 councils that are responsible for healthcare.^5^ Hence, general conclusions on national factors across countries can be difficult.

The second point of critique is that national-level factors might not be relevant for all types of LVCs. Some national-level actions might be used for a specific LVC but will not make any difference for another one depending on how the specific LVC is governed. We have found considerable variation between factors influencing the use of LVC where one factor such as insurance type may lead to an increased use of one LVC practice and decreased use of another.^3^ This makes it problematic to draw general conclusions on national level factors influencing all LVC practices.

 A third point of critique is that the study is based on interviews with 18 experts in the authors’ network in three countries. The experts were chosen trough the Choosing Wisely network and asked about their experiences of “key factors affecting LVC.” This rather limited sample makes it difficult to draw firm conclusions. To understand the generalizability of the findings, it would be helpful to know to what extent this sample is representative of a broader population of experts in the field, perhaps particularly those outside the Choosing Wisely network. It seems likely that other experts would have identified alternative factors. Furthermore, it is difficult to determine the extent to which the experts’ experiences are grounded in empirical reality and to what extent they were articulating more subjective beliefs. This begs the question: to what extent are their opinion-based assessments consistent with facts, assuming there is sufficient research available? Generalizability tends to be compromised with convenience sampling since it entails a risk for biased data.

## Critical Comments on the Findings

The findings by Verkerk et al largely overlap with those of our international scoping review but there are also noteworthy differences.^3^ Fear of malpractice litigation was reported in many studies included in the scoping review.^6-8^ However, fear of malpractice was not found to be a relevant factor in a Swedish interview study with primary care physicians,^4^ suggesting a national-level factor that might differ among countries. Verkerk et al suggest solutions for this fear on an individual level such as strategies to convince the individual physicians that their fear is unfounded. We miss solutions to fear of malpractice on a national or a system level (rather than individual) as the study focuses on national level factors.

Verkerk and colleagues’ described system factors: payment structure and the impact of industry is similar to what we have identified.^2^ Verkerk et al^2^ concluded that LVC use often increases when payment structures emphasize volume over value and fee-for-service payments. These factors’ influence on LVC use was complex and depended on the specific type of LVC practice.^3^ For instance, having private health insurance was related to receiving more of certain LVC practices,^9,10^ while being uninsured or being insured via Medicaid influenced other LVC practices.^11^ While we agree that the financial system influences the use of LVC, there are complex challenges related to changing such systems. For-profit organizations in healthcare do not seem to reduce the use of LVC whereas the organizational norms are more influential.^12^ Furthermore, whenever cost cuts are encouraged, there is a risk that more expensive evidence-based practices will be reduced whereas less expensive LVC practices will be increased. Costs related to LVC are mainly related to low cost procedures.^13^ In addition, many LVCs are low value to some patients and evidence-based for others, making it difficult to cease payment for specific practices since it is still vital for some patients to receive them.

The impact of the pharmaceutical and medical device industry was perceived as a powerful influence through promoting the use of potentially unnecessary care.^2^ Three US-based studies in our scoping review also mentioned how direct marketing to consumers concerning drugs and treatments^14^ and promotion of prostate cancer screening by healthcare companies^15^ or the pharmaceutical industry^8^ were all related to the use of LVC. Interestingly, we did not find any studies from any other countries than the United States that reported industry impact as a factor influencing patients’ LVC use. Thus, differences between countries might exist and drawing conclusions from all three countries might not be relevant.

The social factors (public and medical culture) Verkerk et al found are also in line with previous findings. They refer to *public culture* in terms of individuals’ assumptions, perceptions, and values that favor more care and new technology, all of which might yield increased LVC.^2^ In our scoping review, patient factors were the most common factors influencing LVC use.^16,17^ Similarly, primary care physicians in our interview study perceived pressure from patients as a reason for LVC use.^4^ With regard to medical culture, Verkerk et al^2^ described that clinicians tend to overestimate the benefits of some practices. This finding is similar to a LVC determinant identified in our scoping review^3^: clinicians’ attitudes toward a given LVC practice influences their use of it.^18^ The medical culture was summarized with the maxim “better safe than sorry” to reduce uncertainty and avoid not doing anything.^2^ We noted the same sort of reasoning, as primary care physicians’ desire to do something for the patients influenced their use of LVC.^4^ However, if the authors agree with this conclusion, there should be a clearer emphasis on strategies to target both the public and the medical culture rather than discussing how to change individuals’ mindsets related to fear of malpractice. Cultures are not only built by information to patients and healthcare professionals but also by, as the authors themselves suggest, protecting professionals from the burden of complaints. We suggest that further research is needed on how to practically do so.

## Challenges and Research Needs

LVC research and practice involve many challenges. For instance, the fact that specific LVC practices should generally not be provided to patients, though these practices are appropriate for *some* patient groups, symptoms, and characteristics (eg, due to age or multimorbidity). For instance, antibiotics are not recommended for upper respiratory tract infections,^19^ but are recommended for other bacterial infections. This complexity of LVC use makes it difficult to develop guidelines and policies that give sufficient direction while also leaving enough freedom for clinicians and units to create their own routines. Thus, reducing LVC use represents a substantial challenge; the aim is not always to abandon a practice altogether.

Another challenge when studying factors that influence LVC use is to avoid studying such factors separately, in isolation from each other. Such an approach neglects the fact that two or more seemingly unimportant factors may combine to create powerful effects. While Verkerk et al stress that the national-level factors have a “synergistic relationship” with each other, this is not explored except for the observation that “especially the industry strengthens the other factors.”^2^ We believe it is crucial to view the use of LVC and interventions to reduce its use in holistic terms, as many factors can be expected to be interdependent. This has implications for selecting appropriate strategies to influence LVC use.

 A third challenge for research is to proceed from studies focusing on describing and understanding the problem (ie, what influences LVC use) to identifying appropriate solutions (eg, policy-related strategies). Selecting the most relevant, effective strategies requires in-depth understanding of current practice and the behaviors, routines, or healthcare systems that need to be changed. However, there is no reproducible evidence-based process for taking predictor variables from a descriptive study and turning them into components of strategies. This process will be influenced by human judgment, which is subject to many biases. However, this is an area where further research is needed. Well-designed intervention studies are required to establish the effectiveness of various strategies to influence various types of LVC use. This type of research implies a shift in focus from understanding factors that influence LVC to understanding how and why various strategies influence its actual use among clinicians.

## Ethical issues

 This research has been approved by the Swedish national ethics review authority.

## Competing interests

 Authors declare that they have no competing interests.

## Authors’ contributions

 All three authors have contributed to the manuscript equally.

## Funding

 This work is funded by the Swedish Research Council for Health, Working life and Welfare (FORTE) (2018-01557).

## References

[1] Brownlee S, Chalkidou K, Doust J (2017). Evidence for overuse of medical services around the world. Lancet.

[2] Verkerk EW, Van Dulmen SA, Born K, Gupta R, Westert GP, Kool RB (2022). Key factors that promote low-value care: views of experts from the United States, Canada, and the Netherlands. Int J Health Policy Manag.

[3] Augustsson H, Ingvarsson S, Nilsen P (2021). Determinants for the use and de-implementation of low-value care in health care: a scoping review. Implement Sci Commun.

[4] Ingvarsson S, Augustsson H, Hasson H, Nilsen P, von Thiele Schwarz U, von Knorring M (2020). Why do they do it? A grounded theory study of the use of low-value care among primary health care physicians. Implement Sci.

[5] Augustsson H, Casales Morici B, Hasson H (2022). National governance of de-implementation of low-value care: a qualitative study in Sweden. Health Res Policy Syst.

[6] Greene SE, Massone R (2015). A survey of emergency medicine residents’ perspectives of the choosing wisely campaign. Am J Emerg Med.

[7] Buist DS, Chang E, Handley M (2016). Primary Care Clinicians’ Perspectives on Reducing Low-Value Care in an Integrated Delivery System. Perm J.

[8] Alber K, Kuehlein T, Schedlbauer A, Schaffer S (2017). Medical overuse and quaternary prevention in primary care - A qualitative study with general practitioners. BMC Fam Pract.

[9] Chan PS, Rao SV, Bhatt DL (2013). Patient and hospital characteristics associated with inappropriate percutaneous coronary interventions. J Am Coll Cardiol.

[10] Schmidt ML, Spencer MD, Davidson LE (2018). Patient, provider, and practice characteristics associated with inappropriate antimicrobial prescribing in ambulatory practices. Infect Control Hosp Epidemiol.

[11] Ladapo JA, Blecker S, Douglas PS (2014). Physician decision making and trends in the use of cardiac stress testing in the United States: an analysis of repeated cross-sectional data. Ann Intern Med.

[12] Greenwood BN, Agarwal R, Agarwal R, Gopal A (2016). The when and why of abandonment: the role of organizational differences in medical technology life cycles. Manage Sci.

[13] Mafi JN, Russell K, Bortz BA, Dachary M, Hazel WA Jr, Fendrick AM (2017). Low-Cost, High-Volume Health Services Contribute The Most To Unnecessary Health Spending. Health Aff (Millwood).

[14] Hines JZ, Sewell JL, Sehgal NL, Moriates C, Horton CK, Chen AH (2014). “Choosing wisely” in an academic department of medicine. Am J Med Qual.

[15] Pickles K, Carter SM, Rychetnik L, Entwistle VA (2016). Doctors’ perspectives on PSA testing illuminate established differences in prostate cancer screening rates between Australia and the UK: a qualitative study. BMJ Open.

[16] Cope AL, Francis NA, Wood F, Chestnutt IG (2016). Antibiotic prescribing in UK general dental practice: a cross-sectional study. Community Dent Oral Epidemiol.

[17] Selby K, Cornuz J, Cohidon C, Gaspoz JM, Senn N (2018). How do Swiss general practitioners agree with and report adhering to a top-five list of unnecessary tests and treatments? Results of a cross-sectional survey. Eur J Gen Pract.

[18] Han PK, Klabunde CN, Noone AM (2013). Physicians’ beliefs about breast cancer surveillance testing are consistent with test overuse. Med Care.

[19] Braman SS (2006). Chronic cough due to acute bronchitis: ACCP evidence-based clinical practice guidelines. Chest.

